# Coding of multisensory temporal patterns in human superior temporal sulcus

**DOI:** 10.3389/fnint.2012.00064

**Published:** 2012-08-28

**Authors:** Tömme Noesselt, Daniel Bergmann, Hans-Jochen Heinze, Thomas Münte, Charles Spence

**Affiliations:** ^1^Department of Biological Psychology, Otto-von-Guericke-Universität MagdeburgMagdeburg, Germany; ^2^Center of Behavioral Brain Sciences, Otto-von-Guericke-Universität MagdeburgMagdeburg, Germany; ^3^Department of Neurology, Otto-von-Guericke-Universität MagdeburgMagdeburg, Germany; ^4^Psychosomatic Medicine, Asklepios Westklinikum HamburgHamburg, Germany; ^5^Department of Neurology, UniversitätzuLübeckLübeck, Germany; ^6^Crossmodal Research Laboratory, Department of Experimental Psychology, University of OxfordOxford, UK

**Keywords:** audiovisual, temporal perception, fMRI, speech, human

## Abstract

Philosophers, psychologists, and neuroscientists have long been interested in how the temporal aspects of perception are represented in the brain. In the present study, we investigated the neural basis of the temporal perception of synchrony/asynchrony for audiovisual speech stimuli using functional magnetic resonance imaging (fMRI). Subjects judged the temporal relation of (a)synchronous audiovisual speech streams, and indicated any changes in their perception of the stimuli over time. Differential hemodynamic responses for synchronous versus asynchronous stimuli were observed in the multisensory superior temporal sulcus complex (mSTS-c) and prefrontal cortex. Within mSTS-c we found adjacent regions expressing an enhanced BOLD-response to the different physical (a)synchrony conditions. These regions were further modulated by the subjects' perceptual state. By calculating the distances between the modulated regions within mSTS-c in single-subjects we demonstrate that the “auditory leading (A_L_)” and “visual leading (V_L_) areas” lie closer to “synchrony areas” than to each other. Moreover, analysis of interregional connectivity indicates a stronger functional connection between multisensory prefrontal cortex and mSTS-c during the perception of asynchrony. Taken together, these results therefore suggest the presence of distinct sub-regions within the human STS-c for the maintenance of temporal relations for audiovisual speech stimuli plus differential functional connectivity with prefrontal regions. The respective local activity in mSTS-c is dependent both upon the physical properties of the stimuli presented and upon the subjects' perception of (a)synchrony.

## Introduction

When observers are confronted with incongruent auditory and visual information, that information is often fused into a congruent multisensory percept. Spatial, semantic, and temporal factors have all been shown to contribute to this perceptual fusion (see e.g., Driver and Noesselt, 2008, for a review). The temporal relationship between inputs from different senses plays a particularly important role in multisensory integration (Köhler, 1947; Dennett, 1991; Spence and Squire, 2003; Kelly, 2005) and the perceived synchrony declines when the audio-visual asynchrony exceeds a certain temporal delay. When simple auditory beeps and visual flashes are being judged, subjects' temporal synchrony window spans approximately 100 ms (Slutsky and Recanzone, 2001; Vatakis and Spence, 2006a) becoming broader/wider when stimuli are more complex (consisting of semantic content; Dixon and Spitz, 1980; McGrath and Summerfield, 1985; Spence and Squire, 2003; Miller and D'Esposito, 2005; Vatakis and Spence, 2006b, see also Vroomen and Keetels, 2010 for review).

Several brain structures have been implicated in the multisensory integration of auditory and visual stimuli. Among them are the superior colliculi (Stein and Meredith, 1993), the superior temporal sulcus complex (STS-c), the intraparietal sulcus (IPS), the insular cortex, the claustrum and prefrontal areas (e.g., Calvert et al., 2000; Bushara et al., 2001; Calvert, 2001; Driver and Noesselt, 2008). Within the STS-c, areas within or close to the upper bank have been identified as key regions governing multisensory integration in both humans (Wright et al., 2003; Beauchamp, 2005a; Noesselt et al., 2007) and non-human primates (Benevento et al., 1977; Desimone and Gross, 1979; Bruce et al., 1981; Hikosaka et al., 1988; Barraclough et al., 2005). Direct neuronal recordings from the superior temporal polysensory (STP) region in monkeys have revealed that neurons can respond to both visual and auditory stimuli in both the upper (Bruce et al., 1981; Hikosaka et al., 1988) and lower banks (Benevento et al., 1977). Barraclough et al. (2005) reported neurons within the STS-c that respond to action-related congruent audiovisual stimulation. When focusing on complex, speech-related animal communication, results from studies in macaques suggest that temporal regions in the macaque brain (including in the STS-c) are activated by audiovisual species-specific vocalizations (Gil-da-Costa et al., 2004; Ghazanfar et al., 2008). In humans, using linguistic stimuli, van Atteveldt et al. (2004) found regions in the STS-c that responded to visually presented letters, spoken single letters, or their combination. As in the study by Wright et al. (2003) employing lip-movements plus spoken syllables, the STS-c response was greatest for audiovisual stimuli. van Atteveldt and colleagues (2004) reported that multisensory enhancement was seen for congruent but not for incongruent stimuli. However, other studies reported enhancements in functional magnetic resonance imaging (fMRI)-responses for incongruent stimuli within STS-c (e.g., van Atteveldt et al., 2007). These findings suggest that the STS-c is involved in the temporal binding of audiovisual stimuli. However, it still needs to be established whether congruent or incongruent audiovisual stimuli elicit a higher fMRI-signal in STS-c, or whether different subregions within the STS-c may differentially code multisensory temporal relations.

Hence, the aim of the present study was to investigate the functional neuroanatomy of the multisensory regions including STS-c and prefrontal cortex when perceiving a temporal (mis-)alignment of ecologically-valid long speech sequences; and to examine whether audiovisual temporal relationships may subdivide multisensory regions functionally. Subjects were shown videos of temporally aligned and misaligned video streams [either auditory leading (A_L_) or visual leading (V_L_) and reported whether those were perceived as being synchronous or asynchronous. Importantly, they also reported changes of perceived timing *during* the presentation of each stimulus. This design enabled us to dissociate those neural processes that were related to perceptual switches and those related to stable perceptual states during the presentation of audiovisual speech sequences. To anticipate, we found differential BOLD-effects for the different temporal percepts (A_L_, V_L_, and synchrony (AV_S_)] within adjacent subregions in human STS-c, plus differential interregional connectivity with prefrontal cortex.

## Methods

**Figure 1:**
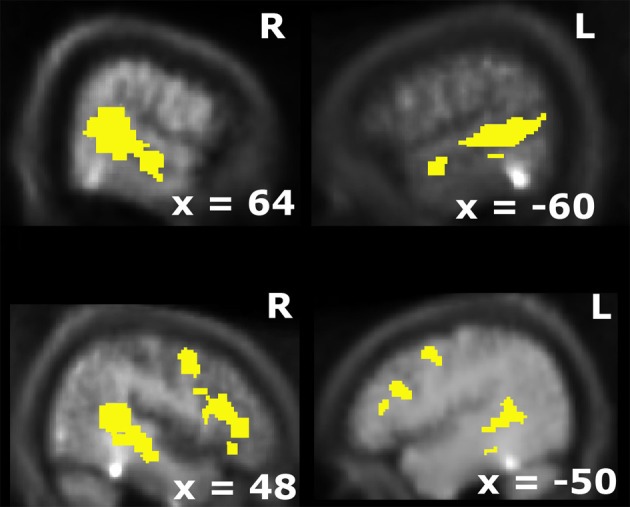
**Overlap of visual and auditory BOLD-modulations for unisensory stimulus presentations (*p* < 0.005; *k* > 10).** This activation map was used as the search volume for the fMRI-analysis in the main experiment.

**Figure 2:**
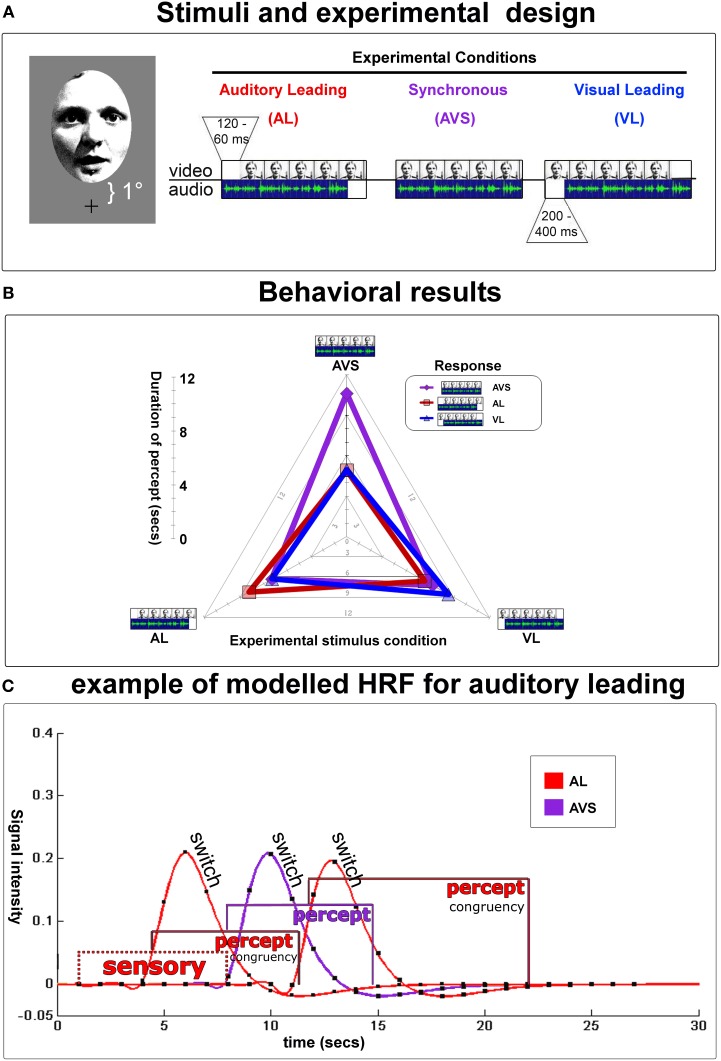
**Experimental design and behavioral results. (A)** Depicts an example of a video-clip presented in three conditions [i.e., auditory leading (top left, temporal lag from 60–120 ms), auditory and video synchronous (top middle), or visual leading (top right, temporal lag from 200–400 ms)]. Auditory and visual lags were determined in a preliminary threshold-determination-experiment. Stimuli were presented at 1° visual angle above fixation (lower boundary) up to 7° (upper boundary). The duration of all 20 video-clips was 23.7 s, the interstimulus interval was 20 s. Partcipants indicated whether they perceived the auditory stream leading, the visual stream leading, or the 2 streams as being synchronous. They were encouraged to report any changes in their perception during the presentation of each video. Note that the physical lag was fixed within each video clip near the individual's synchrony/asynchrony-threshold. **(B)** Radar graph depictsmean durations (time from one keypress to the next) of subjects' (a)synchrony-percepts for each experimental condition during fMRI-scanning: perceptual states were longest when perception of (a)synchrony was congruent with physical stimulation. Therefore, in the fMRI-analysis, hemodynamic response functions (HRF) could be specifically modeled and extracted for each stable percept and perceptual switches using a mixed model (see below). **(C)** An example trial modeled with hemodynamic response functions for an auditory leading-stimulus (A_L_). Gamma-curves depict perceptual switches/decisions, whereas box-car functions illustrate the sensory procesing prior to the first decision and perceptual states. Purple curves stand for AV_S_, red for A_L_. Note that each box-car function was individually specified based on the trial-by-trial inter-button-press duration.

A temporal-threshold experiment was conducted prior to scanning, to account for any individual differences in temporal perception. By choosing bistable stimuli for each subject we maximized the number of trials per condition during the fMRI-experiment (see below). Subjects (*n* = 14, 7 female) were placed in a dark, sound-attenuated chamber after providing written informed consent in accord with local ethics. They had to report the perceived synchrony or direction of asynchrony of auditory and visual information of video sequences by pressing one of three buttons (thereby indicating A_L_, AV_S_, V_L_). Importantly, subjects could change their judgements *during* each video presentation. The stimuli consisted of 20 video clips (length 23.7 s), depicting the face of a trained female speaker reading sentences (see Figure 1). Stimuli were randomized with MATLAB 6.1 and presented using Presentation 9.11 (Neurobehavioral Systems, Inc., CA). Initially, 20 synchronous sequences plus 80 temporally shifted sequences were presented (−130 ms, −60 ms (A_L_) and 200 ms/400 ms (V_L_), 20 video clips each, see Figure 2A). These asynchronies for threshold-determination were chosen in accord with previous reports (Dixon and Spitz, 1980). For the fMRI-experiment, those stimuli were chosen for each subject that had a similar number of synchrony and asynchrony judgments (called near-threshold below).

### fMRI-data acquisition

fMRI-data was acquired on a whole body Siemens 3 T Trio-scanner (Siemens, Erlangen, Germany) using a circular-polarized whole-head coil (BrukerBioSpin, Ettlingen, Germany). Subjects performed the same task as they had outside the scanner, reporting their responses with their right index, middle, and ring finger. Within the scanner subjects were presented three conditions: near-threshold V_L_, near-threshold A_L_ plus the AV_S_ condition. All other stimulus parameters were kept as in the behavioral experiment outside the scanner except for the following: first, a baseline period of 20 s was introduced after each video clip. Second, eye movements were monitored using an fMRI-compatible infrared recording system (Kanowski et al., 2007) plus evaluation software (PupilTracker, HumanScan, Erlangen, Germany). The eye movement data was analysed with MATLAB 6.5. Third, before the main fMRI-experiment, a functional localizer was run in which only unimodal auditory or unimodal visual stimuli from the videos were presented (331 volumes covering the whole head, TR 2 s, TE 30 ms, flip 80°, resolution 64 × 64 × 32 at 3.5 × 3.5 ×4 mm). The derived overlapping audio-visual activation map was then used to identify candidate multisensory areas (see below). Fourth, subjects wore earplugs; perceived loudness and balance were adapted individually to ensure easy comprehension of the auditory speech sequences despite the scanner noise. The stimuli were presented using MR-compatible, electrodynamic headphones (MRconfon, Magdeburg, Germany).

During the main experiment functional volumes were collected in four sessions (331 volumes each, covering the whole head, TR 2 s, TE 30 ms, flip 80°, resolution 64 × 64 × 32 at 3.5 × 3.5 ×4 mm). Additionally, for anatomical localization an inversion-recovery EPI was acquired (TR 2 s, TE 30 ms, TI:1450 ms, resolution 64 × 64 × 32 at 3.5 × 3.5 ×4 mm, same slice orientation and distortions as the functional volumes). The first five volumes from each session were excluded from further analysis. The remaining volumes were acquisition-corrected to the first acquired slice of each volume, motion-corrected, normalized at 2 mm^3^ voxel size and smoothed (6 mm), using SPM2 (Wellcome Department of Cognitive Neurology, London, UK).

### Group-level statistics

After pre-processing the data from a localizer run were modeled with two box-car functions convolved with the hemodynamic response function (HRF) for the auditory and visual trials. For the localizer runs, blocks were compared to the baseline during which no stimulus was present (*p* < 0.005; *k* > 10). An audiovisual mask (i.e., overlap of unisensory visual and auditory activations) was computed to identify candidate multisensory structures (see Figure 1; cf. Beauchamp et al., 2004b; Beauchamp, 2005b; Noesselt et al., 2007; Szycik et al., 2008).

Next, all experimental conditions were modeled with the HRF with variable durations when appropriate (mixed model; see Figure 2C). In particular, 21 conditions were defined in a mixed model: three perceptual switches (subjects' button press, event-related), three perceptual states (time after button press, variable block) and the initial stimulation (time before the first button press, variable block) for every stimulus condition (A_L_, V_L_, and AV_S_). To test condition effects, linear contrasts were used for each subject and condition and masked inclusively with the audiovisual overlap from the functional localizer. The resulting contrast images were applied to perform random effects second-level analyses. The statistical parametric maps of the *t*-statistics at each voxel were thresholded at *p* < 0.05 (small-volume-corrected) and the spatial extent threshold was set at *k* > 5 voxels.

The following contrasts were computed: First, we identified regions that responded to physical synchrony and asynchronous conditions. Second, we identified regions that showed differential fMRI-signals for perceived synchrony vs. asynchrony conditions. Finally, we computed interaction effects for differential perceptual states with identical physical stimulation (i.e., asynchronous vs. synchronous percepts separately for A_L_, V_L_, and AV_S_ stimulation).

### Single-subject statistics

We also analysed the data from individual subjects in order to confirm our group-level results and to test the interaction between stimulation and percepts formally. We identified for each subject regions within STS-c using the identical contrasts as in the group analysis above: for A_L_ stimulation: veridical A_L_ percept > non-veridical synchronous percept; for AV_S_ stimulation: veridical synchrony percept > both non-veridical percepts; for V_L_ stimulation: veridical V_L_ percept > non-veridical synchronous percept. Subject-specific regions of interest (ROI) were identified by searching for significant clusters of the three contrasts of interest within subregions of the STS-c (anatomical criterion) which expressed unisensory responses to both modalities (additional functional criterion). We extracted the beta-weights of all experimental conditions from these three local maxima for each subject and tested whether these local maxima would express significantly different results across stimulations. Note that this analysis is non-trivial and provides additional information, since any BOLD-modulation of different perceptual states to the AV_S_-stimulation was left unspecified in the A_L_ and V_L_ stimulation contrasts and vice versa.

### Analysis of interregional connectivity

Complementary to the analysis of local modulations of the BOLD-response we investigated the effects of interregional connectivity in the context of perception of AV_S_, V_L_, and A_L_ as described above (Friston et al., 1997). We seeded our analyses in the subject-specific local maxima in STS-c and analyzed which other regions showed enhanced functional coupling in the context of A_L_ percepts in the A_L_ condition (relative to non-veridical synchronous percept in the A_L_ condition), in the context of V_L_ percepts in the V_L_ condition (relative to the non-veridical synchronous percept in the A_L_ condition) and in the context of synchronous percepts in the synchronous condition (relative to the non-veridical asynchronous percept in the synchronous condition) using a model with 21 regressors (see above) plus the physiological response and the psychophysiological interaction as two additional regressors (see e.g., Noesselt et al., 2007 for a similar approach) to reveal differential functional interregional connections in the psychological context of synchronous or asynchronous percepts. Three models were calculated separately for each STS-local maximum (corresponding to veridical A_L_-percepts, veridical V_L_-percepts, and veridical AV_S_ percepts).

Differential group-level effects were calculated with an analysis of variance (ANOVA) pertaining the three PPIs from the three connectivity analysis.

### Analysis of consistent patterning of subregions

Finally, distances between single subject maxima in STS-c were computed and analysed to reveal any systematic anatomical distribution of subjects' local maxima for the A_L_, V_L_, and AV_S_ representation. For this we used a three step approach: normalization of MNI-coordinates, calculation of distances by subtracting the normalized MNI-coordinates and calculation of Euclidian distances in three-dimensional space. In particular, for the calculation of distances, the MNI coordinates (in millimeters) of the three contrasts and their respective local maxima were scaled by adding the maximum negative value to all coordinates of one dimension so that all values were positive. This procedure was applied for the *y* and *z* extension/dimension; *x* coordinates were converted into their absolute value. Second, coordinate values of the same dimension but different local maxima were subtracted from each other (A_L_/V_L_ minus synchrony and A_L_ minus V_L_). Finally, we computed Euclidean distances for the difference measures: following Pythagoras' Theorem, difference values of the *x* and *y* dimension (cathetuses) were squared and added together and the resulting value (hypotenuse) added to the squared *z* dimension difference. The square roots of the resulting values (again hypotenuse) represent the reported distances between voxels.

## Results

### Behavioral results

The results of the behavioral experiment outside the scanner revealed that subjects' judgments became more consistent with stimulation as the audiovisual delay increased. For the auditory stream leading condition, the mean delay for near-threshold stimuli was 105 ms (±35 ms) while for the visual stream leading condition it was 227 ms (±47 ms). Inside the scanner, subjects again judged temporal relations of the video clips while fMRI-data were acquired. The eye-movement data were analysed using both deviations from fixation and eye blinks (Kanowski et al., 2007). Three subjects who showed extensive eye movements or blinking were excluded from further analysis. In the remaining 11 subjects, neither “real” eye movements nor eye blinks showed any differential effect across the experimental conditions (i.e., eye movements < 1°).

During each video subjects (*n* = 11) switched 5.72 (2.34 SD) times toward a “congruent” perceptual state [i.e., one during which perception and the physical stimulus were identical] vs. 3.97 (2.0) times toward a non-veridical one. Moreover, subjects maintained veridical percepts for 9.13 (3.38) s on average, whereas non-veridical percepts lasted 6.04 (2.02) s (see Figure 2B for length of stable durations as a function of the stimulus type). This allowed for an unbiased mixed model design (see Figure 2C and Kleinschmidt et al., 1998; Dosenbach et al., 2006 for similar approaches).

### Neuroimaging results

#### Voxel-based group results

First, we computed candidate multisensory structures (i.e., the overlap of activation patterns found with unisensory visual and auditory stimuli before the main experiment; see Beauchamp et al., 2004b; Noesselt et al., 2007; Szycik et al., 2008, for similar approaches). These candidate multisensory structures comprised of bilateral superior temporal sulcus, bilateral anterior insula extending into prefrontal cortex plus bilateral premotor cortex.

When comparing stable perceptual states with switches we found stronger fMRI-responses in bilateral STS-c and lateral prefrontal cortex for the maintenance of perceptual states relative to switches whereas perceptual switches engaged posterior parietal regions plus anterior cingulate in accord with earlier studies (e.g., Heekeren et al., 2008). Since perceptual switches did not significantly modulate voxels within temporal regions, we then focused on the experimental effects of the different stimulus types and of stable perceptual states (i.e., inter-response intervals) within multisensory regions.

**Figure 3:**
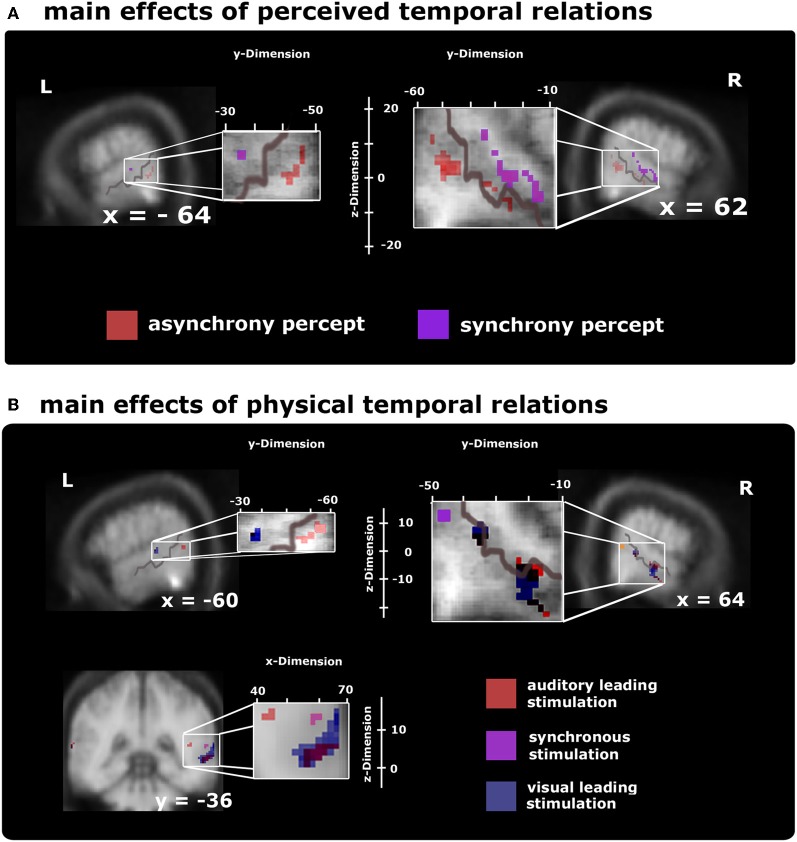
**Voxel-based group BOLD-effect of subjects' stable perceptual states (time from one keypress to the next, upper row) and the effects of the different stimulus types (lower row) within audiovisual activation maps (as defined by the overlap of unisensory stimuli) thresholded at ***p*** < 0.05 (small-volume-corrected).** Note that the distribution of time-sensitive regions differed in the left and right hemisphere, with the left hemisphere showing a more widespread pattern than the right hemisphere (as evidenced by the formatting). **(A)** Comparison of synchrony > asynchrony percepts collapsed over stimulus type (purple spots) highlights modulations reaching from posterior to middle STS-c. Adjacent regions within STS-c were also found to be relevant for stable asynchrony percepts > synchronous ones (red spots; additionally, the asynchrony > synchrony percepts-contrast produced significant modulations in prefrontal areas; not shown, see Table 1). **(B)** Differential BOLD-responses for the three stimulus types collapsed over peceptual state show significant effects at the right posterior STS-c (purple spots; plus premotor regions; not shown, but see Table 2) for synchronous relative to asynchronous stimulation; at both STS-c (blue spots) and prefrontal areas (not shown) for visual leading relative to synchronous stimuli, and at left posterior STS-c and right anterior/posterior STS-c (red spots plus modulations at precentral gyrus and prefrontal areas; not shown, see Table 2) for auditory leading relative to synchronous stimulation.

First, comparisons of AV_S_ vs. (V_L_+A_L_) perceptual states (collapsed over stimulus types) revealed modulations in adjacent subregions of bilateral multisensory STS-c, in right insular cortex, and in bilateral prefrontal areas (see Figure 3A and Tables 1A,B); note that both asynchronous and synchronous perceptual states modulated regions within STS-c, whereas only asynchronous perceptual states additionally modulated the anterior insula and prefrontal cortex (see Table 1). Second, comparisons of the physically AV_S_ minus (V_L_+A_L_) stimuli (regardless of perceptual states) revealed right-lateralised modulations in middle and posterior STS-c plus prefrontal cortex (see Figure 3B, purple spots). A_L_ and V_L_ stimuli (relative to synchronous stimuli; see Figure 3B, red and blue spots, respectively) showed enhanced BOLD-responses in bilateral STS-c, prefrontal cortex, and anterior insula (see Tables 2A–C for local maxima). Please note, that the time-related modulations are more widespread in the left hemisphere, which might be a reason for the left-sided dominance of synchronous representation reported in other studies (e.g., Miller and D'Esposito, 2005; Marchant et al., 2012).

**Table 1:** **Local maxima (*p* < 0.05, *k* > 5 small-volume-corrected) for (A) synchrony minus asynchrony perception within multisensory regions (see Figure 3A, purple spots) and (B) asynchrony minus synchrony percepts (see Figure 3A, red spots) collapsed across physical stimulation**.

**Anatomical structure**	**Hemisphere**	**Cluster size (voxels)**	***t*-value**	**MNI coordinates**		
				***x***	***y***	***z***
**A.SYNCHRONY PERCEPT > ASYNCHRONY PERCEPT**						
**Temporal Lobe**						
Anterior STS	R	96	4.95 (0.001)	60	−22	−2
Anterior STS	−	22	3.68 (0.005)	62	−10	−10
Posterior/middle STS	R	32	2.84 (0.01)	48	−38	8
Posterior/middle STS	L	13	3.47 (0.007)	−58	−34	−4
**B. ASYNCHRONY PERCEPT > SYNCHRONY PERCEPT**						
**Temporal Lobe**						
Posterior/middle STS	R	206	5.63 (0.000)	66	−34	−6
Posterior/middle STS/MTG	R	10	2.98 (0.007)	58	−40	−8
Posterior/middle STS	R	14	2.92 (0.008)	54	−44	18
Posterior/middle STS	L	17	3.56 (0.006)	−66	−50	2
**Frontal Lobe**						
Anterior insula	R	14	2.66 (0.01)	42	36	−10
Prefrontal cortex	R	644	10.09 (0.000)	56	24	22
Prefrontal cortex	L	9	2.44 (0.02)	−54	30	14

MNI, Montreal Neurological institute; L, left; R, right.

**Table 2:** **Local maxima (*p* < 0.05, *k* > 5 small-volume-corrected) for (A) AV_S_ minus (A_L_+V_L_) stimulation within multisensory regions (see Figure 3B, purple spots); (B) V_L_ minus synchrony stimulation (see Figure 3B, blue spots); and (C) A_L_ minus synchrony stimulation (see Figure 3B, red spots) collapsed across perceptual states**.

**Anatomical structure**	**Hemisphere**	**Cluster size (voxels)**	***t*-value**	**MNI coordinates**		
				***x***	***y***	***z***
**A. PHYSICAL SYNCHRONY > PHYSICAL ASYNCHRONY**						
**Temporal cortex**						
STS	R	9	2.39 (0.03)	54	−46	14
STS	R	8	2.31 (0.04)	62	−50	10
**Frontal lobe**						
Prefrontal cortex	R	14	2.92 (0.008)	50	36	12
**B. PHYSICAL VISUAL LEADING ASYNCHRONY > PHYSICAL SYNCHRONY**						
**Temporal lobe**						
Anterior STS	R	370	3.81 (0.001)	64	−20	−12
Middle STS	L	9	3.05 (0.005)	−68	−38	14
Middle STS	L	14	2.95 (0.007)	−60	−30	8
**Frontal lobe**						
Prefrontal cortex	R	41	2.78 (0.01)	38	18	26
Prefrontal cortex	L	11	2.73 (0.01)	−46	20	28
Anterior insula	R	8	2.99 (0.006)	50	42	2
Anterior insula/IFG	L	41	3.88 (0.001)	−36	38	−16
**C. PHYSICAL AUDITORY LEADING ASYNCHRONY > PHYSICAL SYNCHRONY**						
**Temporal Lobe**						
Anterior STS	R	177	3.55 (0.002)	62	−14	−8
Posterior/middle STS	R	122	3.32 (0.002)	54	−46	−2
Middle STS	L	12	4.07 (0.001)	−68	−38	14
Posterior STS	L	57	2.86 (0.009)	−54	−54	8
**Frontal Lobe**						
Precentral gyrus	R	17	3.27 (0.003)	44	0	40
Precentral gyrus	R	7	2.47 (0.02)	48	6	44
Anterior insula/IFG	L	6	3.31 (0.002)	−36	40	−18
Prefrontal cortex	L	19	2.46 (0.02)	−46	22	24

MNI, Montreal neurological institute; L, left; R, right.

**Figure 4:**
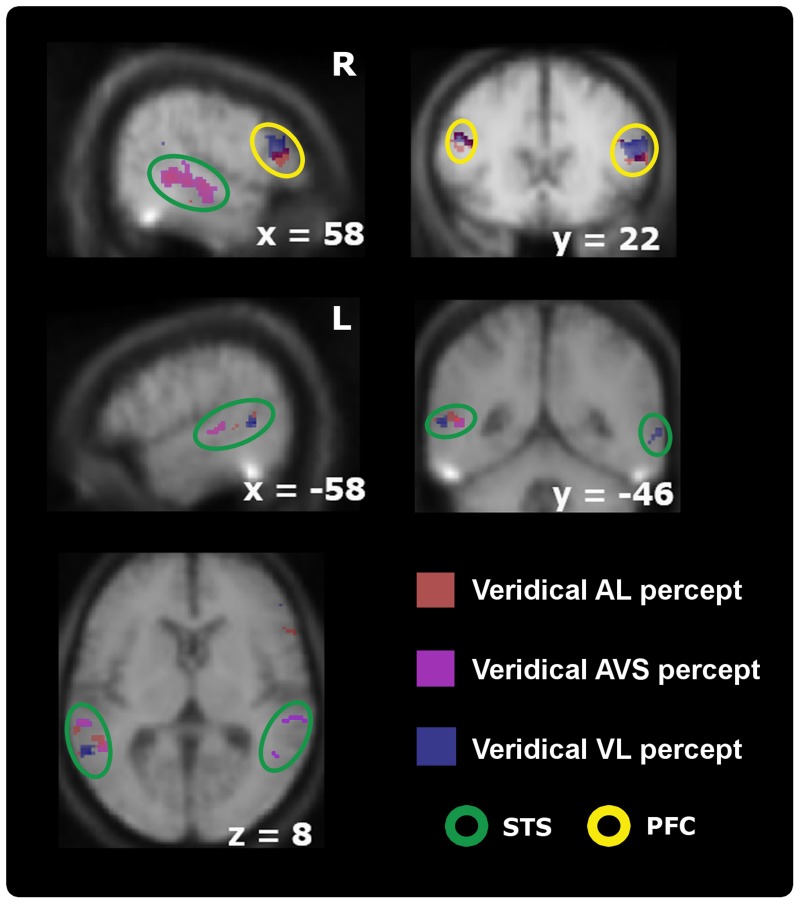
**Activation maps comparing participants' stable veridical percepts (i.e., identical with the physical stimulus) against non-veridical percepts within audiovisual regions thresholded at *p* < 0.05; *k* > 5 (small-volume-corrected).** Whereas the right-sided STS-c is only modulated by synchronous > asynchronous percepts (orange spots, upper left panel) when processing synchronous stimuli, the results also show higher activations for asynchronous judgments during asynchronous stimulation [both auditory (red spots) and visual leading (blue spots); represents coherence] compared to synchronous judgments during asynchronous stimulation (incoherence) within prefrontal regions (top right) and the left STS-c region (middle and lower row). Note that within this area analysis revealed distinct spots for each veridical percept. Prefrontal regions were only modulated by veridical percepts during asynchrony stimulation (see Table 3).

Finally, we compared different perceptual states separately for each stimulus type (and not collapsed across stimulus type as above). Note that these stimulus-type-specific comparisons were designed to reveal perceptual effects for identical physical stimuli. Differential non-overlapping BOLD-modulations were again found in anterior insula, prefrontal cortex, and STS-c; with only asynchronous perceptions expressing higher activations in the insula and prefrontal cortex (see Figure 4, plus Tables 3A–C). Within STS-c, distinct regions for synchronous and asynchronous perceptions were observed as a function of stimulus type. BOLD-modulations for AL and VL conditions (veridically perceived as asynchronous) enclosed a region with an enhanced BOLD-response for veridically perceived AVS stimuli within the left hemisphere (see Figure 4, middle row and lower left panel). In the right hemisphere, regions within the STS-c responded to veridically perceived AVS and VL stimuli (see Figure 4, middle and bottom row). We also investigated whether we would find modulations in the fMRI-signal for the main effects of stimulus type, perception and perceptual states for each stimulus type outside the multisensory ROI. However no significant modulations were observed (p_FWE−corrected_ < 0.05, since we did not have any *a priori* hypothesis).

**Table 3:** **Local maxima (*p* < 0.05, *k* > 5 small-volume-corrected) for (A) Auditory leading minus synchrony percepts during A_L_ stimulation within multisensory regions (see Figure 4, red spots); (B) synchrony minus (A_L_ + V_L_) percepts during AV_S_ stimulation (see Figure 4, purple spots); and (C) V_L_ minus synchrony percepts during V_L_ stimulation (see Figure 4, blue spots)**.

**Anatomical structure**	**Hemisphere**	**Cluster size (voxels)**	***t*-value**	**MNI coordinates**		
				***x***	***y***	***z***
**A. COHERENT AUDITORY LEADING PERCEPT > COHERENT SYNCHRONY PERCEPT**						
**Temporal lobe**						
Posterior/middle STS	L	57	2.62 (0.01)	−52	−42	4
Posterior/middle STS	L	18	2.96 (0.01)	−64	−38	10
Posterior/middle STS	L	6	2.85 (0.01)	−64	−36	−8
Middle STS	R	8	3.43 (0.005)	−54	−30	−14
Anterior/middle STS	R	15	2.95 (0.007)	−64	−42	12
**Frontal lobe**						
Anterior insula	L	20	3.64 (0.001)	−32	28	−6
anterior insula	R	66	6.52 (0.000)	42	32	−6
Precentral gyrus	R	86	3.98 (0.002)	48	10	40
precentral gyrus	L	28	4.67 (0.000)	−36	8	60
Precentral gyrus	L	11	3.41 (0.006)	−40	8	38
Prefrontal cortex	R	191	3.25 (0.003)	54	28	12
Prefrontal cortex	L	12	2.97 (0.01)	−50	6	44
**B. COHERENT SYNCHRONY PERCEPT > COHERENT ASYNCHRONY PERCEPT**						
Anterior STS	R	447	4.24 (0.001)	62	−16	−4
Anterior STS	L	62	4.05 (0.002)	−62	−24	4
Posterior STS	R	5	2.66 (0.02)	48	−52	10
Posterior/middle STS	L	29	3.28 (0.007)	−50	−46	8
**Frontal lobe**						
Anterior Insula/Prefrontal	L	14	2.63 (0.01)	−34	40	−14
**C. COHERENT VISUAL LEADING PERCEPT > COHERENT SYNCHRONY PERCEPT**						
**Temporal lobe**						
Posterior STS	R	18	3.5 (0.002)	64	−50	2
Posterior STS	R	5	2.41 (0.02)	62	−50	14
Posterior STS	L	29	3.47 (0.002)	−58	−50	8
**Frontal lobe**						
Anterior insula	R	72	4.77 (0.000)	44	40	−10
Anterior insula	L	20	2.71 (0.008)	−34	30	−2
Precentral gyrus	R	21	3.42 (0.002)	42	8	46
Prefrontal cortex	R	451	3.76 (0.001)	50	24	24
Precentral cortex	L	134	3.56 (0.002)	−42	16	26

MNI, Montreal Neurological institute; L, left; R, right.

#### Single-subject region-of-interest approach

Because of the possible anatomical differences between subjects within the STS-c (Ochiai et al., 2004), a ROI analysis was performed within single subjects to confirm and extend voxel-based group-level responses to physical and/or perceptual (a)synchrony.

**Figure 5:**
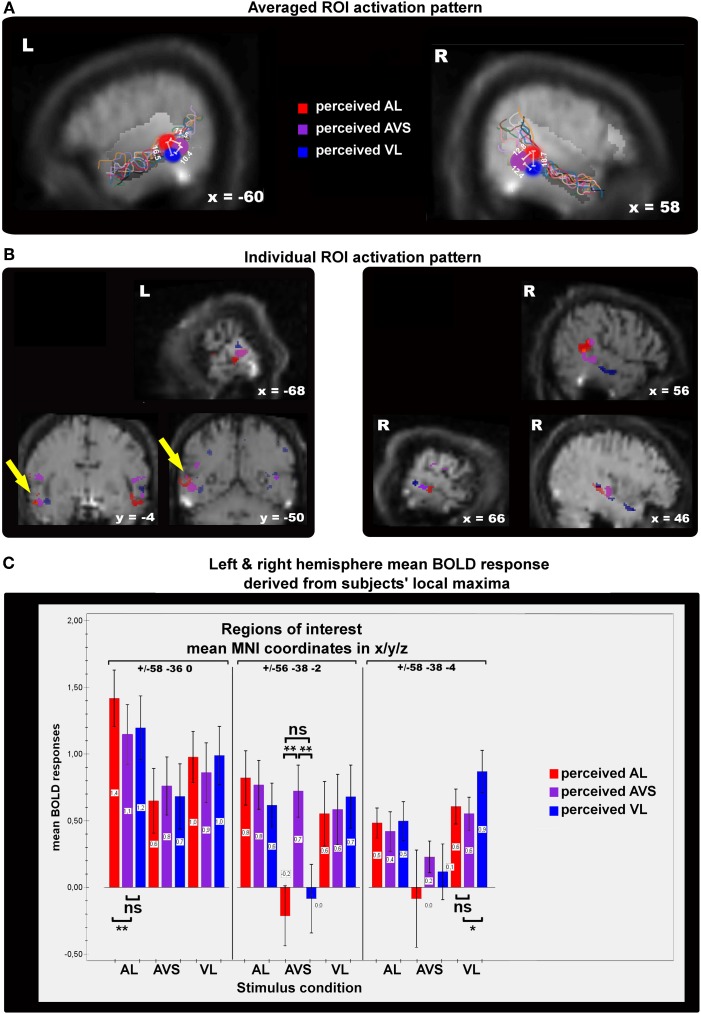
**Panels showing the results of single-subject analyses.** The contrasts displayed here represent subjective perceptions that were congruent with physical stimulation > incogruent perceptions for auditory/visual leading (AL,VL, red/blue spots) and synchronous stimuli (AVS, purple spots). **(A)** The colored spots indicate average local maxima (11 subjects) of areas that express higher activations for veridical percepts (see main text for contrast definitions) than for non-veridical ones within the STS-c region (region of interest). The white lines and their corresponding numbers display averaged distances in millimeters from one activation spot to the two others (see “Methods” section for details). Note that asynchrony spots are always more distant from each other than from synchrony activation. Colored lines show the individual anatomical curvatures of STS-c of the all subjects after normalization. **(B)** The middle row depicts the activation maps of three individual subjects for the above-described contrasts. Note that synchrony spots (purple) are enclosed by two asynchrony spots [blue and red spots; see also distances in panel **(A)**]. Such activation patterns were found in both left and right hemispheres. **(C)** Mean beta-weights (proportional to % signal change) for the local maxima in panel **(A)** were collapsed over hemispheres. Bars show the height of the BOLD-effect (y-axis) for each stable percept [auditory leading (red bars), synchronous (orange bars), and visual leading (blue bars)] for the three stimulus types (auditory leading, visual leading, and synchrony, x-axis) within each each of the local maxima shown in panel **(A)** [auditory leading percept maximum (left graph section), synchrony percept maximum (middle section of graph), and visual leading percept (right graph section)]. BOLD-responses to asynchrony percepts *within* asynchrony percept maxima were always higher (outer left and right bars) than to any other percept for the different stimulus types. Within the synchrony percept maximum BOLD-responses to synchrony percepts were higher than asynchrony percepts whenever synchronous video clips were presented.

For this ROI analysis, three differential temporal percepts were evaluated for each subject with the following contrasts: veridical (asynchronous) minus non-veridical synchronous perception for A_L_ and V_L_ speech stimuli; plus synchronous minus asynchronous perception for AV_S_ stimulus trains. This analysis was again applied within subjects' audiovisual masks. Mean beta weights responses (proportional to percent signal change) for the subjects' perceptual states in every experimental condition were assessed for the three (a)synchrony areas and their respective local maxima. (Note, that these local maxima were identified by conducting comparisons of a limited number of perceptual states, regardless of any other differential effects between conditions. Thus, the analysis of BOLD-effects reported below, will provide additional information concerning the overall response patterns within the STS-c-subregions):

We extracted the beta weights for all perceptual states (3 states × 3 stimulus types) from the three local maxima within STS-c and conducted a 2 × 3 × 3 × 3 repeated measures ANOVA with the factors of hemisphere, type of (a)synchrony area, percept, and stimulus type (see Figure 5B). As no effect of hemisphere was found [*F*_(1, 10)_ < 1; n.s.], beta weights averaged over hemispheres are displayed in Figure 5C. Interaction effects occurred between type of area, percept, and stimulus type [*F*_(8, 80)_ = 3.1; *p* < 0.01] suggesting that, within each (a)synchrony area, beta weights change as a function of the subjects' percept and stimulus type. Main effects were observed for type of (a)synchrony area [*F*_(2, 20)_ = 4.9; *p* < 0.05] and percept [*F*_(1.33, 13.26)_ = 10.9; *p* < 0.01]. Although *post-hoc t*-tests showed no significant effects, responses within the “V_L_ areas” were lower than in the other two areas. BOLD responses to synchronous stimuli were significantly lower than to asynchronous stimuli [*t*_(20)_ = −3.53; *p* < 0.01]. Interaction effects occurred between hemisphere and type of area [*F*_(2, 20)_ = 8.04; *p* < 0.01], type of area and percept [*F*_(4, 40)_ = 3.48; *p* < 0.05], type of area and stimulus type [*F*_(1.73, 17.3)_ = 9.17; *p* < 0.01], percept and stimulus type [*F*_(1.7, 17.06)_ = 4.7; *p* < 0.05].

Further analysis of the ANOVA-data (*post-hoc t*-tests) revealed that for each stimulus category, subjects' BOLD responses were highest when a veridical judgment was made. Within the “A_L_ area” (red), the mean BOLD response was highest when subjects perceived an A_L_ stimulus as A_L_ (veridical percept). The according beta weight differed statistically from the two other beta weights and their respective perceptual states [*t*_(10)_ = 3.12; *p* < 0.05], whereas the beta weights of the non-veridical percepts did not differ statistically from each other. The same pattern of results was also observed for the AV_S_ region (yellow) [*t*_(10)_ = 4.76; *p* < 0.001] and V_L_ percepts (blue) [*t*_(10)_ = 2.72; *p* < 0.05]. Since, in the AV_S_ area, veridical responses were not significantly different from BOLD-responses for other stimulus types, this region may serve additional sub-functions on top of the maintenance of synchrony perception. In general, these ROI-results reaffirm the functional micro-compartmentalization of the STS-c found in the voxel-based group results into areas specialized for the perception of distinct audiovisual temporal patterns.

#### Interregional connectivity of STS-c-regions

**Figure 6:**
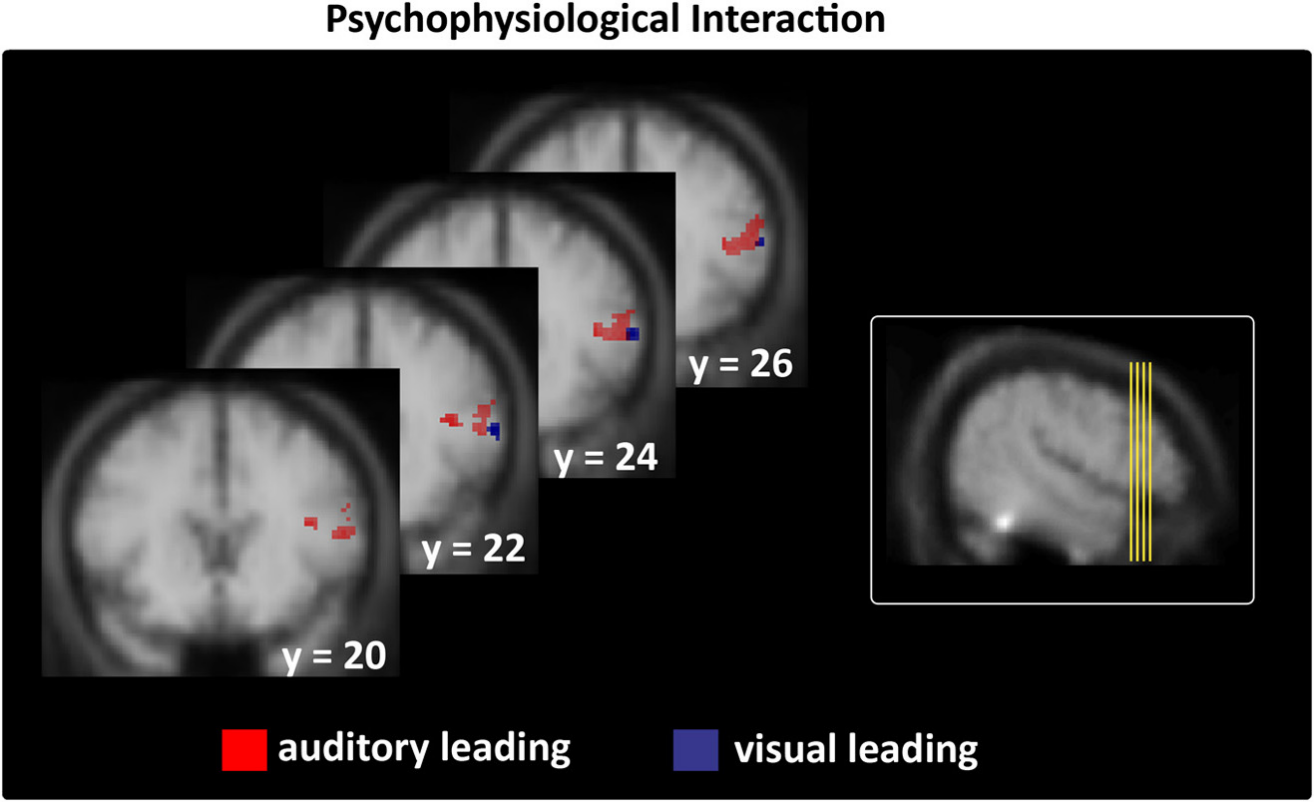
**Interregional connectivity of subjects' stable veridical percepts (i.e., identical with the physical stimulus) during asynchronous stimulation thresholded at *p* < 0.05; *k* > 5 (small-volume-corrected).** Left column: right prefrontal regions only expressed stronger coupling with temporal regions in the context of veridical asynchronous > non-veridical synchronous perceptions during A_L_ and V_L_ stimulation (see Table 4 for all maxima). Right column shows the origin of the brain sections depicted on the left on a lateral group mean view.

Moreover, we assessed whether the subregions within STS-c that consistently expressed differential local activity (see Figure 5) would also be functionally linked to other multisensory regions. We used the assumption-free “psychophysiological interaction” (PPI; Friston et al., 1997) and seeded our analysis in subject-specific STS-c maxima. We analysed whether the strength of functional coupling of these adjacent STS-c-regions with other multisensory regions would differ. We found that both A_L_ and V_L_-regions in bilateral STS-c showed a significantly stronger coupling with right prefrontal regions than did the AV_S_-region (see Figure 6 and Table 4). Moreover, synchronous patches with the middle STS-c expressed a stronger functional connection with posterior STS-c regions in the left hemisphere, whereas asynchronous patches showed a stronger coupling with posterior STS-c in the right hemisphere (see Table 4).

**Table 4:** **Local Maxima (*p* < 0.05, *k* > 5 small-volume-corected) of interregional connectivity in the context of (A) veridical synchrony percepts (relative to non-veridical asynchrony percepts) during AV_S_ stimulation; (B) veridical auditory leading percepts (relative to non-veridical synchrony percepts) during A_L_ stimulation; (C) veridical visual leading percepts (relative to non-veridical synchrony percepts) during V_L_ stimulation**.

**Anatomical structure**	**Hemisphere**	**Cluster size (voxels)**	***p*-value**	**MNI coordinates**		
				***x***	***y***	***z***
**A. PSYCHOPHYSICAL INTERACTION OF SYNCHRONY PERCEPTS**						
**Temporal regions**						
Posterior STS	L	31	4.32 (0.001)	−54	−54	12
**B. PSYCHOPHYSICAL INTERACTION OF AUDITORY LEADING PERCEPTS**						
**Temporal regions**						
Anterior STS	R	73	3.57 (0.001)	64	−12	−8
Posterior STS	R	22	3.32 (0.001)	−50	−46	16
**Frontal regions**						
Middle/inferior frontal gyrus	R	168	2.94 (0.002)	40	22	16
precentral gyrus	L	54	2.68 (0.005)	−48	−2	46
**C. PSYCHOPHYSICAL INTERACTION OF VISUAL LEADING PERCEPTS**						
**Temporal regions**						
Posterior STS/STG	R	44	2.42 (0.009)	52	−46	0
**Frontal regions**						
Inferior frontal gyrus	R	21	2.51 (0.008)	58	22	14

#### Spatial configuration of STS-c-subregions

In addition, we evaluated whether the spatial configuration of the identified sub-regions within bilateral STS-c showed a systematic spatial distribution across subjects: the analysis revealed that perceived asynchrony (A_L_ or V_L_) and synchrony modulated distinct regions along the STS-c which were situated adjacent to one another (with asynchrony enclosing synchrony modulations). For every subject, this specific pattern differed in its position along STS-c but occurred regularly (see Figure 5A for average, Figure 5B for illustrative subjects). Distances between the areas modulated by an interaction of stimulus type and perception were calculated. We found that, on average, the local maxima of the “A_L_” and “V_L_ areas” were situated closer to “synchrony areas” (12.1 and 11.1 mm) than to each other (17.6 mm).

A 2 × 3 repeated measures ANOVA with the factors hemisphere and distance showed an effect of distance [*F*_(2, 20)_ = 10.2; *p* < 0.001]. The distance between the “asynchrony areas” was statistically different from their respective distance to the “synchrony area” [A_L_: *t*_(10)_ = 3.77; *p* < 0.05; V_L_: *t*_(10)_ = 3.40; *p* < 0.05]; the distances between the asynchrony areas and the “synchrony area” were similar [*t*_(10)_ = 0.63; *p* = 0.55]. There was no effect of hemisphere [*F*_(1, 10)_ < 1; n.s.], nor any interaction between hemisphere and distance [*F*_(2, 20)_ < 1; n.s.].

## Discussion

The present study investigated the neural basis of both the processing of physical properties and subjective perception of the temporal relationship between auditory and visual speech stimuli, thereby pinpointing the functional neuroanatomy of audiovisual temporal processing and perception in multisensory cortex in humans. We found that sub-regions within the superior temporal sulcus have a distinct response pattern during the maintenance of perceptual states and for the processing of physical stimulus differences regardless of subjects' perceptual state. Within lateral prefrontal regions and anterior insula only the perception of asynchrony was consistently linked to an increase in BOLD-response. A ROI-based single-subject analysis corroborated and extended this pattern: three subregions within the STS-c showed a differential response for the different physical stimuli (AL, VL, and AVS). Responses were further enhanced if subjects' perceptual states were congruent to the physical stimulus being presented. Further, analyses of interregional connectivity suggest that during the perception of asynchronous stimuli AL and VL regions within the STS-c are coupled more strongly to lateral prefrontal regions, whereas connectivity within posterior STS-c was lateralized with stronger connections of the middle with posterior STS-c in the left hemisphere for synchrony patches and with posterior STS-c in the right hemisphere for asynchronous patches. Finally, analysis of the anatomical patterning of these regions suggests that they are distributed regularly within the STS-c with a synchrony region being enclosed by asynchrony regions.

Previous neuroimaging studies have reported that the STS-c (among other structures) is involved in audiovisual temporal processing and synchrony perception (Calvert, 2001; Macaluso et al., 2004; Miller and D'Esposito, 2005; Dhamala et al., 2007; Noesselt et al., 2007; Stevenson et al., 2010; Marchant et al., 2012; see Driver and Noesselt, 2008, for a review). However, most of these studies investigating the crossmodal binding of semantically meaningful stimuli (Calvert et al., 2000; Calvert and Campbell, 2003; Macaluso et al., 2004) did not separate task- and perception-related effects; their reported modulations may therefore reflect a mixture of stimulus-, decision-, and perception-related processing.

Previous research (Miller and D'Esposito, 2005; Stevenson et al., 2010) reported effects of the temporal fusion of short AV-syllables using event-related fMRI. Stevenson and his colleagues (2010) reported functional subregions within STS-c, that preferentially processed asynchronous or synchronous speech. Miller and D'Esposito (2005) reported left-hemispheric modulations within STS-c for perceptual fusion and right hemispheric effects for perceptual segregation. However, the differences in stimulus materials used in the various conditions may explain the different activation maps reported there. Nonetheless, while we did not find lateralized effects of the local fMRI-signal, our interregional connectivity analysis revealed a lateralized pattern, that accord with Miller and D'Esposito.

Other studies have investigated the effects of audiovisual timing with streams of simple stimuli: Calvert et al. (2001) investigated multisensory interactions using simple synchronized and desynchronized audiovisual stimulus sequences. Synchronous or asynchronous bimodal inputs showed non-linear enhancements or suppressions (respectively) of BOLD-responses in multisensory areas, including STS-c, plus frontal regions. Noesselt et al. (2007) reported effects in contralateral STS-c for the processing of lateralized non-semantic synchronous audiovisual stimuli, but did not report effects for asynchronous audiovisual stimuli. In a related study, Marchant et al. (2012) observed left-sided synchrony representations in left STS-c. Meanwhile, van Atteveldt et al. (2004, 2007) identified lateral temporal areas (PT, STP, and STS-c) as major integration sites whenever audiovisual grapheme-morpheme pairs were being processed. While the intensity of modulations increased in auditory areas for semantically congruent conditions, the location of modulations within the STS-c changed as a function of the temporal distance/delay between vision and sound: asynchrony was predominantly processed at the eccentricity of the STS-c activation pattern, whereas smaller temporal delays were related to the activation's core region. However, no effect of synchrony was reported for synchronous audiovisual letters in the STS-c and the reported activations for different audiovisual lags overlapped substantially.

In the present study, asynchronous percepts engaged the posterior STS-c, the anterior insula, and the prefrontal cortex bilaterally. Our results accord with previous imaging studies on temporal asynchrony which reported right-sided effects within the STS-c, supplementary motor areas (Miller and D'Esposito, 2005) and prefrontal (MFG, IFG) cortices (Bushara et al., 2001; Dhamala et al., 2007) in the perception of asynchrony. Our findings corroborate previous results and suggest that audiovisual prefrontal areas and the STS-c are functionally linked during the maintenance of the perception of audiovisual asynchrony. There is also corroborating anatomical evidence that the STS-c is reciprocally linked to prefrontal regions (see e.g., Yeterian et al., 2012). We speculate that the perception of asynchronous percepts may be more demanding than synchrony perception and requires the on-line updating of two separate working memory representations in prefrontal cortex with input from the STS-c. Alternatively, the separation of auditory and visual input may be processed by prefrontal cortical regions (in line with the notion of a hierarchical multisensory processing model, see e.g., Noppeney et al., 2010) and fed back into the STS-c. Future research in non-human primates or in humans using transcranial magnetic stimulation/transcranial direct current stimulation is needed to disentangle these two possibilities.

Most remarkably of all, our results indicate that the multisensory superior temporal sulcus complex (mSTS-c) can be further differentiated into subregions that process particular audiovisual temporal patterns. Anatomical studies in non-human primates that have investigated the anatomical texture of TPO (the likely homologue to the human STS-c; Beauchamp, 2005a) have provided evidence for three caudal-to-rostral subdivisions within this region (Cusick et al., 1995). Those subdivisions are distinct in terms of their chemoarchitecture. Seltzer and Pandya (1991) provided evidence that TPO consists of cytoarchitectonic subdivisons of which particularly the rostral part is directly connected to the insula. Further chemoarchitectonic results support the view that the upper bank of TPO in the rhesus monkey contains several different anatomical and functional zones (Padberg et al., 2003). They demonstrated that within those distinct neurochemical/connectional modules the STS-c shows a patchy organization of connections toward other cerebral regions. Those patches within the STS-c may have functional relevance. In a functional imaging study, Beauchamp et al. (2004a) reported that STS-c can be parcellated into unisensory auditory, visual, and multisensory patches. Our imaging analysis extends these findings and reveals distinct multisensory patches along the STS-c that encode separate audiovisual temporal patterns when the synchrony/asynchrony of continuous speech is being judged. Given that the identified synchrony patches lie in-between auditory- and visual-leading audiovisual patches, these modulations build up a chronological array that suggests the existence of a “time line.” Moreover, another publication (Fairhall and Macaluso, 2009) also reported a modulation of the fMRI-signal due to attention within middle but not posterior STS-c, when subjects processed congruent audiovisual speech, thereby suggesting a large-scale segregation of the STS-c along the anterior-posterior axis (though asynchronous representations seem to be more variable; see Tables 1–3). Moreover, Marchant et al. (2012) investigated the correspondence of an audiovisual behavioral benefit on BOLD-modulations in the cerebrum and found significant effects in middle but not posterior STS-c for synchronous stimulus trains. The results from our study—revealing an interaction effects in middle STS-c specific for temporal patterns and their perception plus an enhanced connectivity with more posterior regions—are in accord with this proposition (though note that our results did not reveal a clear anterior-posterior distinction for the main effects of physical vs. perceptual states). Finally, our results could be applied to nonhuman primates to enable more invasive measures [combined with fMRI (see Tsao et al., 2006)] to identify the pathways and neural mechanisms involved. A study in non-human primates on audiovisual face-voice integration (Ghazanfar et al., 2008) reported enhanced coupling of STS-c-neurons with auditory areas when processing audiovisual stimuli (Schroeder et al., 2008). Our results would predict the existence of distinct patches within mSTS-c that may differentially engage unisensory cortices via feedback connections (Driver and Noesselt, 2008).

In conclusion, we found a distinct pattern of modulations within mSTS-c reflecting an interaction between perceptual state and the physical properties of audiovisual speech stimuli. Our data therefore suggest that there is an aligned spatial representation of audiovisual temporal patterns parcellating the multisensory STS-c in humans, with differential functional connections to multisensory prefrontal regions.

### Conflict of interest statement

The authors declare that the research was conducted in the absence of any commercial or financial relationships that could be construed as a potential conflict of interest.
